# DMEK after penetrating keratoplasty: cohort with DMEK grafts and descemetorhexis larger than full-thickness graft

**DOI:** 10.1007/s00417-022-05641-6

**Published:** 2022-04-05

**Authors:** F. A. Steindor, J. Menzel-Severing, M. Borrelli, S. Schrader, G. Geerling

**Affiliations:** 1 Department of Ophthalmology, University of Düsseldorf, Moorenstraße 5, 40225 Düsseldorf, Germany; 2 Department of Ophthalmology, Pius-Hospital, Carl Von Ossietzky University, Oldenburg, Germany

**Keywords:** Descemet Membrane Endothelial Keratoplasty (DMEK), Penetrating keratoplasty (PK), Graft failure, Graft oversizing, Descemet stripping

## Abstract

**Purpose:**

The study aims to evaluate visual outcome, central corneal thickness, and rebubbling rate in a cohort with oversized DMEK grafts after failed penetrating keratoplasty (PK). The unique feature of the study is a descemetorhexis diameter larger than the full-thickness graft, i.e., peripheral to the PK interface.

**Methods:**

A monocentric, retrospective evaluation of all patients with endothelial graft failure after PK treated with an oversized DMEK graft and descemetorhexis outside of the PK interface (i.e., in host tissue) between January 2015 and July 2019 at the Department of Ophthalmology at the University of Düsseldorf (Germany) was performed.

**Results:**

Eleven eyes of 10 patients were identified. Mean age was 69 years. On average (arithmetic mean ± standard deviation), 1.7 ± 1.0 previous PKs have been performed per eye in this cohort. The mean time between last PK and DMEK was 10.1 ± 7.3 years (range 2 to 23 years). In all cases, the graft diameter exceeded the diameter of the previous PK and descemetorhexis was performed in host tissue, that is, peripheral to the graft-host interface. Rebubbling was performed in 18.2% of the patients (*n* = 2 eyes) because of central graft detachment. Mean central corneal thickness showed a statistically significant improvement at 5.3 ± 3.5 months after surgery from 688.23 ± 151.01 to 527.75 ± 88 µm (*p* = 0.002).

Visual acuity increased significantly by 5 lines from 1.24 ± 0.5 logMAR (range from 0.5 to 2) to 0.73 ± 0.76 logMAR (range from 0.1 to 2) within 3 months (*p* = 0.006). Excluding patients without visual potential and transplant failure, visual acuity improved significantly by 8 lines (*p* < 0.001), and stayed stable until the last follow-up at 15.1 ± 11.4 months (range 6 to 39 months, *p* < 0.001, *n* = 8) after surgery.

**Conclusion:**

DMEK can be successfully used to treat endothelial cell failure after PK, and can provide good postoperative results with regards to visual acuity. This study shows that stripping of Descemet’s membrane (DM) peripheral to the PK interface is surgically feasible. Overlapping, larger DMEK grafts with more endothelial cells can be used without increasing rebubbling rates and may potentially improve long-term graft survival.

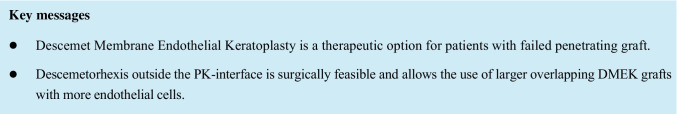

## Introduction

The introduction of Descemet Membrane Endothelial Keratoplasty (DMEK) into clinical practice in 2006 has considerably advanced surgical treatment of Fuchs endothelial corneal dystrophy, bullous keratopathy, and other corneal endothelial diseases [1]. This has led to an increasing number of corneal transplantations and earlier surgical intervention in corneal endothelial diseases [2, 3].

In comparison to penetrating keratoplasty (PK), DMEK leads to faster visual rehabilitation, does not always require corneal sutures, has a lower surgically induced astigmatism, and lower risk of immunological rejection. Therefore, DMEK could be considered the technique of choice in patients with endothelial graft failure after PK, e.g., if uncorrected or spectacle-corrected vision was good prior to graft failure, and/or in patients with higher risk of rejection, and/or in patients with ocular surface disorders [4]. This approach seems suitable for visual rehabilitation in eyes with failed PK but absent stromal scarring and higher, irregular corneal astigmatism. It may — due to its lower trauma to the ocular surface and reduced risk of immune reaction — be preferable in eyes with severe ocular surface disease or for pain relief in eyes with bullous keratopathy but limited or no any visual potential [5].

Several studies have shown feasibility and favorable results of posterior lamellar keratoplasty (ie., Descemet stripping automated endothelial keratoplasty (DSAEK) or DMEK) in patients with failed PK [4, 6–11]. Regardless of the advantages of DMEK in patients after PK, high rates of graft detachment have been reported and constituted the most common reason for graft failure in a recent case series [10]. A recently published retrospective study by Pasari et al. showed a higher air reinjection rate when the diameter of the DMEK graft was oversized relative to the previous PK. Rebubbling rates were reported to be 53% when the DMEK diameter was oversized, 27% when diameters of both grafts matched, and 33% when the DMEK diameter was smaller to that of the previous PK. Stripping was performed within the interface of previous PK and host’s tissue [4]. Here we report clinical data from 11 patients undergoing DMEK after failed PK. The unique feature of the cohort in this study is the fact that all patients were treated with DMEK grafts that were larger in diameter than the original PK and descemetorhexis was performed peripheral to the graft-host interface.

## Methods

A monocentric, retrospective review of all patients with endothelial graft failure after PK treated with oversized DMEK grafts and descemetorhexis outside of the PK interface in host tissue between January 2015 and July 2019 at the Department of Ophthalmology at the University of Düsseldorf (Germany) was performed. The study was approved by the ethics committee at the University of Düsseldorf (study number: 2020–1014).

All procedures were performed under general anesthesia. Donor tissues were organ cultured and provided from either LIONS eye bank (Düsseldorf, Germany) or ETB-Bislife (Beverwijk, The Netherlands). Tissue preparation was performed immediately before the actual operation by the surgeons without any marking. Paracentesis and an anterior chamber (AC) maintainer were used to achieve complete air fill of the AC. The host’s DM was scored within the host’s tissue, that is, peripheral to the graft-host interface using a Price hook. In a recent published case report by the authors successful stripping per continuitatem without tearing the DM interface of host and PK donor can be seen in a supplemental video [12]. Air was subsequently removed; the graft was inserted using a glass shooter, unfolded, and fixed with an 80%/20% mixture of air and sulfur hexafluoride gas taking up approximately 80% of the volume of the anterior chamber. Postoperative supine position was respected until the intraocular gas had been completely absorbed.

The diameter of all DMEK grafts exceeded the diameter of previous PKs. Mean diameter of the previous PK was 7.73 ± 0.2 mm (range from 7.25 to 8 mm). Diameter of the DMEK grafts was 8.25 mm. Mean overlap was 0.52 ± 0.2 mm (range from 0.25 to 1 mm). Individual sizing characteristics are shown in Table 1.

**Table 1:** Sizing characteristics of DMEK grafts

	PK diameter in mm	DMEK diameter in mm	Descemetorhexis diameter in mm	Overlap in mm
1	7.7	8.25	8.75	0.55
2	8	8.25	8.75	0.25
3	7.75	8.25	8.75	0.5
4	8	8.25	8.75	0.25
5	7.75	8.25	8.75	0.5
6	7.7	8.25	8.75	0.55
7	7.75	8.25	8.75	0.5
8	7.25	8.25	8.5	1
9	7.7	8.25	8.75	0.55
10	7.7	8.25	8.75	0.55
11	7.7	8.25	8.75	0.55
Mean	7.73	8.25	8.73	0.52
SD	0.2	0	0.08	0.2

Postoperatively, dexamethasone 1 mg/ml and ofloxacin 3 mg/ml unpreserved eye drops were administered five times daily for 1 week. Antibiotic drops were then discontinued and dexamethasone drops tapered monthly to once daily. Prednisolone acetate was administered systemically (1 mg/kg per day) postoperatively and tapered every 3 days over 3 weeks.

Successful DMEK was defined as a clear cornea at 3 months after transplantation. Primary graft failure was defined as an absence of corneal clearing after the surgery or recurrence of corneal edema with loss of visual acuity within 3 months after surgery. Graft rejection was defined as new signs of graft rejection like anterior chamber flare/cells, Khodadoust line, or keratic precipitates which could not be explained otherwise.

Patient characteristics, number of previous corneal transplants in the eye receiving a DMEK, size of previous PK, size of DMEK graft including size of descemetorhexis, intraoperative and postoperative complications including rebubbling or graft rejection, best corrected visual acuity (BCVA), and central corneal thickness (CCT) were collected. CCT was measured using Scheimpflug tomography (Pentacam®, Oculus, Wetzlar, Germany). Data for endothelial cell density (ECD) were available in three cases and were measured with specular microskopie (E-3000, Tomey, USA). Baseline donor endothelial cell density was measured with phase-contrast microscopy (Eclipse TE200, Nikon, Japan). All data were recorded and analyzed using Microsoft Excel (2011 for Mac OS). For statistical significance testing, paired *t*-test (BCVA and CCT) was used. The *p*-values under 0.05 were considered statistically significant.

## Results

Eleven eyes from ten patients were included in this study. Seven patients were female, four male. Mean age was 69 ± 7.5 years (standard deviation; range 59 to 80 years). Main indication for the first PK graft was Fuchs endothelial corneal dystrophy (*n* = 5 eyes, 45.5%), followed by keratoconus (*n* = 4 eyes, 36.4%). Two of patients were operated because of corneal scarring (*n* = 2 eyes, 18.2%). In one patient due to herpetic keratitis, in the other one the reason for corneal scarring remained unclear.

Mean time between last PK and DMEK was 10.1 ± 7.3 years (range 2 to 23 years). On average, 1.7 ± 1.0 previous PKs had been performed per eye (range 1 to 4). The cause of endothelial cell failure after PK was late endothelial cell failure in eight eyes (72.7%) and immunologic rejection in three eyes (27.3%).

More than one quarter of all patients had preoperative comorbidities limiting preoperative and postoperative visual acuity (*n* = 3 eyes, 27.3%). All of these patients suffered from glaucoma. From these patients, one had previous filtration surgery, and one patient multiple cyclophotocoagulation.

Surgery was uneventful in all cases; in particular, there was no documented intraoperative adverse event related to the previous PK such as reopening of the stromal interface. Almost all patients were treated with DMEK only. One patient underwent simultaneous phacoemulsification and IOL implantation.

**Fig. 1:**
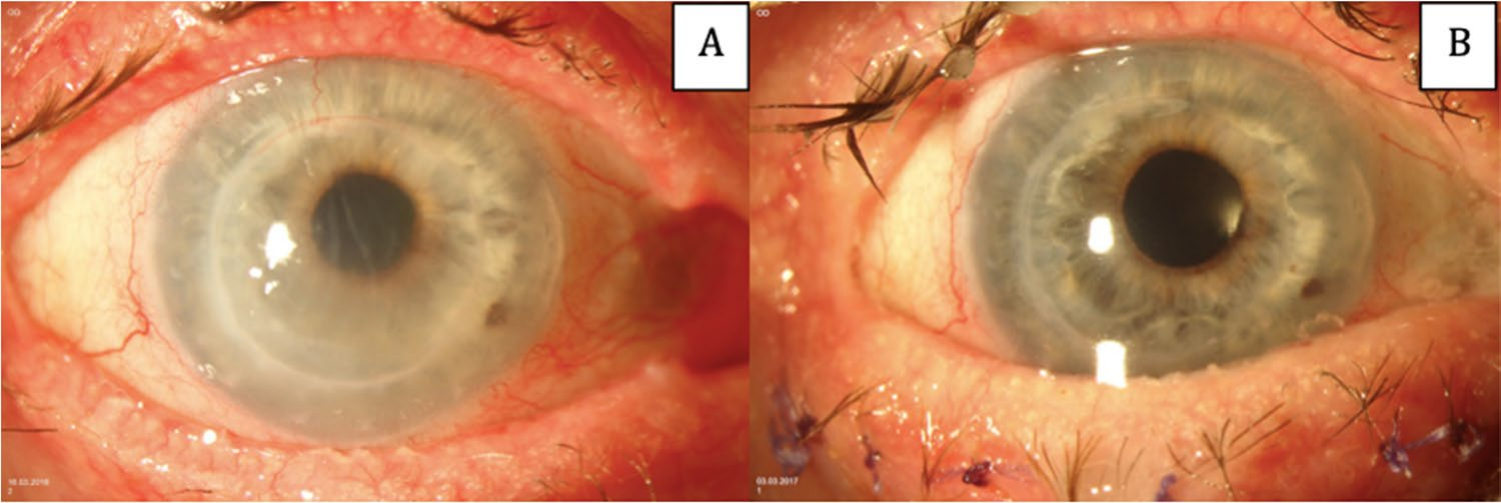
Preoperative (**A**) and postoperative (**B**) findings in a patient with DMEK surgery after failed PK. Preoperatively, the cornea shows stromal edema, Descemet’s membrane folds, and beginning vascularization of the graft. At 2 months postoperatively with an attached DMEK graft, reduced edema, and clear cornea. The presented patient was 76 years old and had initially been transplanted because of advanced keratoconus. Besides his failed PK the patient suffers from ocular surface disorders because of atopy. Visual acuity improved from 1.2 to 0.1 logMAR after 2 months

Mean follow-up was 16.7 ± 11.6 months (range from 6 to 39 months). Figure 1 shows an example of the preoperative and postoperative findings of a patient with DMEK surgery after failed PK. In two patients, significant graft detachment developed with involvement of the optical zone. One could be successful treated with reinjection of gas; another patient refused further intervention after first gas reinjection, particularly rebubbling. Immunologic graft failure with anterior chamber flare/cells and keratic precipitates was observed in one patient 2 months after surgery, which was not successfully treated with intensified oral and local steroid medication combined with mycophenolate mofetil (9.1%). This patient had previously received a total of four grafts in both eyes and had developed bilateral immunologic reaction in the past after PK.

### Visual acuity

**Fig. 2:**
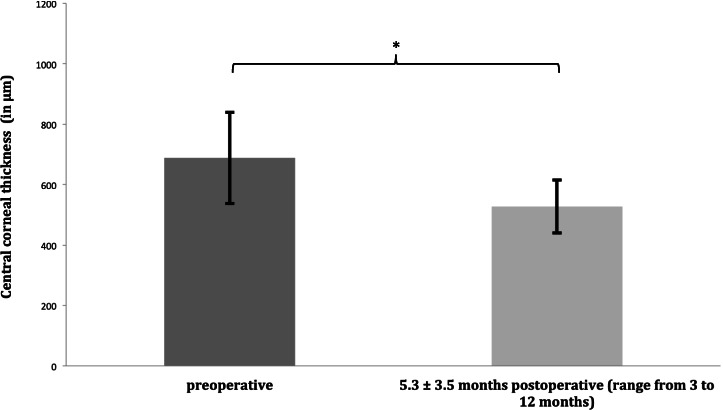
BCVA with failed transplants and patients without visual potential: Visual acuity increased significantly (asterisk = *p* < 0.05) by 5 lines from 1.24 ± 0.5 logMAR (range from 0.5 to 2) to 0.72 ± 0.75 logMAR (range from 0.1 to 2) at last follow-up 16.7 ± 11.6 months, postoperatively (range from 6 to 39 months). Data are mean ± standard deviation (*n* = 11)

Figure 2 shows the postoperative course of visual acuity in all patients (*n* = 11). Visual acuity increased by 5 lines from 1.24 ± 0.5 logMAR (range 0.2 to 2) to 0.73 ± 0.76 logMAR (range 0.1 to 2) within 3 months (*p* = 0.006) and remained stable until last follow-up 16.7 ± 11.6 months postoperatively (range from 6 to 39 months, 0.72 ± 0.75 logMAR, *p* = 0.006). Due to patients without visual potential and failed transplants, standard deviation is high.

Disregarding failed transplants and patients without visual potential with central visual field defects because of glaucoma BCVA improved substantial. Eight eyes were included in this analysis. Mean preoperative BCVA in this cohort was 1.06 ± 0.48 logMAR (range 0.5 to 2). Visual acuity significantly improved by 3 months by a mean of 8 lines (*p* < 0.001) to 0.31 ± 0.2 logMAR (range 0.1 to 0.7) and remained stable without significant chances by 6 months at 0.35 ± 0.19 logMAR (range 0.1 to 0.7, *p* = 0.215) and by 12 months at 0.29 ± 0.12 logMAR (range 0.1 to 0.4, *p* = 0.439). Both improvements were significant compared to preoperative BCVA (*p*_6 months/12 months_ < 0.001). Mean BCVA at the last follow-up 15.1 ± 11.4 months postoperatively (range from 6 to 39 months) was 0.29 ± 0.12 logMAR (range 0.1 to 2, *p* < 0.001 compared to baseline).

### Central corneal thickness

**Fig. 3:**
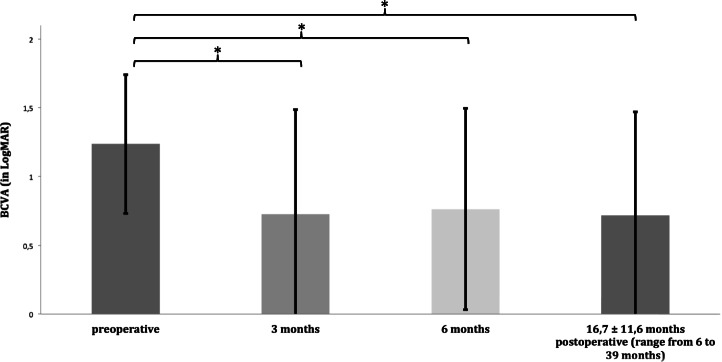
Comparison of central corneal thickness showed a significant (asterisk = *p* = 0.002) reduction by 160.875 µm at a mean of 5.3 months after surgery. Data are mean ± standard deviation (*n* = 8)

Data of central corneal thickness were available for 72.7% of the patients during follow-up (*n* = 8 eyes; Fig. 3). One patient with graft detachment, who refused rebubbling, was not included in this analysis, because CCT was only measured 35 months after surgery. Mean CCT significantly decreased from 688.23 ± 151.01 preoperatively to 527.75 ± 88 µm at 5.3 ± 3.5 months postoperatively (range 3 to 12 months, *p* = 0.002).

### Rebubbling

Rebubbling was performed in two cases to treat graft detachment involving the optical zone (18.2%). One of these patients developed persistent graft detachment with primary graft failure despite rebubbling, but refused further interventions, particularly the necessary repeated rebubbling.

### Endothelial cell density

Endothelial cell density could only be reliable measured with a specular microscope in three cases. Baseline donor endothelial cell density was 2555 ± 73 cells/mm^2^ (range from 2482 to 2628). Mean endothelial cell density was 1375 ± 415 cells/mm^2^ (range from 897 to 1634) 17.3 ± 9.9 months (range from 6 to 24 months) after surgery.

## Discussion

DMEK is a therapeutic option for patients with endothelial graft failure after PK. Although the speed of visual recovery compared to regular DMEK is slower, the advantages are considerable. These include faster visual rehabilitation, lower surgically induced astigmatism, lower risk of immunological rejection, and absence of long-lasting corneal sutures [4]. Known disadvantages of repeated PK such as intraoperative suprachoroidal hemorrhage, persistent epithelial defects, wound leakage, or loose sutures are avoided [13]. In this cohort, we observed a significant improvement in BCVA and central corneal thickness after surgery.

Despite these potential advantages, the surgical technique for DMEK in patients after PK regarding DM stripping and diameter of the graft is still under debate. Stripping could be performed inside the old PK graft, at the interface or in the recipient, peripheral to of the previous PK. Each variant is associated with specific advantages and disadvantages. A smaller descemetorhexis by stripping inside the previous PK followed by a smaller DMEK graft is associated with lower numbers of transplanted endothelial cells. Stripping within the interface leads to adapted wound edges may improve tissue healing, but has the disadvantage of risking opening the stromal interface and may lead to dehiscence or even perforation. Decemetorhexis in the host tissue (i.e., outside the PK graft) favors the use of oversizing DMEK graft but carries the risk of aggravated intraoperative course, e.g., due to strong attachments at the interface and possibly lead to piecemeal removal. A larger graft — compared to the host cornea — and thus more donor EC might help endothelial survival [14]. Indeed it has already been shown that in DSAEK a larger graft diameter is associated with a significantly reduced graft failure rate [15].

A recently published retrospective study by Pasari et al. showed a higher rebubbling rate when the diameter of the DMEK was oversized compared to the previous PK. The authors speculated that this was due to posterior irregularities in the transition zone of host and PK graft. However, in contrast to our study, in the report by Pasari et al. stripping was performed within the edge of the PK wound [4]. It has previously been shown that overlapping of the DMEK graft over host’s endothelium leads to a higher detachment rate and more often results in rebubbling [16].

**Fig. 4:**
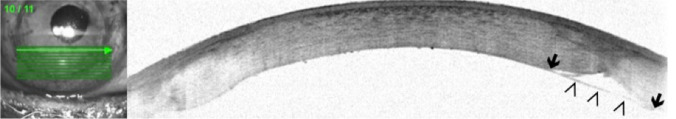
Anterior segment optical coherence tomography 2 weeks after DMEK of the same case shown in Fig. 1. The DMEK graft extends beyond the stromal interface of the previous PK, where it is not fully attached (arrowheads). Because the DMEK graft was attached outside the interface, no rebubbling was performed (arrows). During further follow-up no complications and no increasing graft detachment were observed

Our study shows that DM stripping outside the PK interface is surgically feasible and allows the stripping of a larger posterior corneal surface. This in turn is the prerequisite for using larger DMEK grafts with more endothelial cells (Fig. 4).

A major limitation of our study is a relatively small cohort of 11 patients, the retrospective aspect of our study, and the lack of ECD measurements for each patient. Therefore, our study cannot prove that prolonged graft survival can be achieved by an oversized graft. Moreover, due to the lack of a control group with grafts of the same size or smaller, it cannot be shown that this surgical technique leads to better results in our hands.

**Fig. 5:**
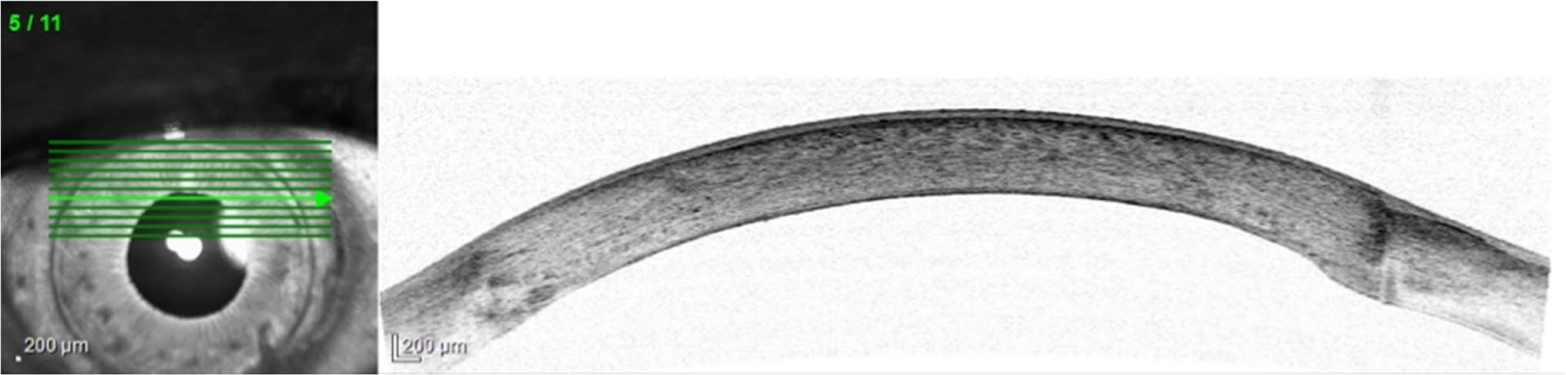
Preoperative anterior segment optical coherence tomography (SPECTRALIS®, Heidelberg Engineering, Heidelberg, Germany). Host-donor interface was slightly thickened posteriorly but without steps. Because of the continuous DM, descemetorhexis was performed successfully in the host tissue beyond corneal host-donor interface

Further studies are required to determine whether preoperative or intraoperative visualization (e.g., anterior segment optical coherence tomography) of the transition zone of the previous PK may help to detect less favorable preoperative conditions (e.g., posterior interface step or dehiscence), which are likely to prevent successful stripping outside of the transition zone and graft attachment (Fig. 5) [17]. An overlapping DMEK graft can be performed successfully especially with a descemetorhexis larger than full-thickness graft. In case of steps between host and previous PK we suggest a graft size big enough to form a tissue bridge to avoid graft detachment.
